# Point cloud registration based on surface feature extraction and an improved Grey Wolf Optimization algorithm

**DOI:** 10.1038/s41598-025-04437-y

**Published:** 2025-06-01

**Authors:** Zimei Tu, Yichen Xie, Jinhua Jiang, Qin Qin

**Affiliations:** School of Intelligent Manufacturing and Control Engineering, Shanghai Polytechnic University, Shanghai, 201209 China

**Keywords:** Electrical and electronic engineering, Mechanical engineering

## Abstract

This study introduces an innovative feature point extraction method combined with an improved Grey Wolf Optimizer (GWO)-based coarse registration approach to address common challenges of low registration accuracy and slow processing speed in point cloud registration. The feature extraction design method begins by projecting the point cloud onto a uniformly segmented sphere. Principal component analysis (PCA) is then employed to compute the curvature change rate of the point set within each patch area. Subsequently, sampling weights are assigned nonlinearly based on the calculated change rates, facilitating effective feature point extraction. The extracted feature points serve as the initial values for the improved gray wolf optimization algorithm, which is employed to refine the registration results. Experimental comparisons conducted on three public datasets demonstrate that the feature extraction method proposed in this study achieves improved accuracy and efficiency. Furthermore, the registration results substantiate that our method outperforms other algorithms with respect to both accuracy and computational efficiency.

## Introduction

Two-dimensional (2D) images are a widely used visual data format. However, the lack of spatial depth information limits their effectiveness in complex structural modeling and geometric analysis. In contrast, three-dimensional (3D) data provide a direct representation of spatial geometry and have increasingly become the mainstream choice for processing real-world scenes. This trend is evident across various domains, including machine vision^1^ reverse engineering^2^ and cultural relics preservation^3^. As a vital representation of 3D data, point clouds can be obtained directly through LiDAR, structured-light scanning, photogrammetry, or other modalities^4^. Raw point cloud data typically require a series of preprocessing and structural analysis steps. Wei^5^ proposed an efficient Wiener filtering enhancement method to improve both point cloud quality and rate-distortion performance. However, due to the non-penetrative nature of laser beams, a single scan rarely yields a complete point cloud^6^. Multiple scans from different viewpoints must be aligned into a unified coordinate system, making point cloud registration a pivotal step in 3D data processing. Point cloud registration typically consists of two stages: coarse registration and fine registration^7^. Coarse registration provides a reliable initial pose for fine registration. If the coarse alignment exhibits a significant deviation, fine registration often fails to achieve satisfactory results. The accuracy of coarse registration not only determines the final quality of the overall registration but also governs the efficiency and stability of subsequent 3D reconstruction^8^. Consequently, high-quality coarse registration methods are crucial for robust 3D point cloud processing.

Coarse registration methods can be categorized into global feature-based and local feature-based approaches^9^. Compared with global feature-based methods, local feature-based techniques are more stable when dealing with occlusions and missing regions^10^. As a result, local feature-based registration has attracted increasing attention. When working with large-scale point clouds, detecting discriminative keypoints in the initial data is especially important^11^.

In the early 21st century, a series of point cloud keypoint detectors was introduced. Sipiran^12^ proposed a three-dimensional Harris detector (Harris 3D, H3D) based on the advantages of the 2D Harris operator, which analyzes local curvature changes in mesh vertex neighborhoods to capture corner-like or sharp features. Zhong^13^ later introduced the Intrinsic Shape Signature (ISS), a novel 3D object recognition descriptor that defines local geometric features for each surface point. Deng^14^ proposed an edge point extraction method based on orthogonal guided rotation sphere (OGRS) for accurate extraction of the boundaries of circular holes, while incorporating normal vector constraints to adapt to point cloud data with curvature variations. However, an evaluation study on point cloud keypoint detectors^15^ indicates that existing approaches often exhibit low repeatability under real-world conditions. Moreover, the need to iterate over all points typically results in high computational cost.

Deep learning methods have achieved efficient feature extraction in the field of 2D image analysis. Zhang^16^ proposed a belief transfer clustering rule for image segmentation and classification, which enhances region-level feature consistency by modeling mutual trust relationships between image patches. Zhao^17^ proposed a Target-Driven navigation method based on deep reinforcement learning (DRL), incorporating a causal intervention mechanism to enhance the agent’s decision-making capability and generalization performance in complex environments. Wang^18^ propose a novel embedded cross framework with a dual-path transformer (ECF-DT) for high-resolution salient object detection. By integrating a dual-path Transformer and a unit fusion module, the framework effectively combines global and local information. In recent years, deep learning-based feature learning methods have attracted extensive attention in point cloud feature extraction. Yu^19^ developed a Point Transformer-based deep learning network PipeSegNet to fully extract global and local features that are independent of resolution issues and scanning quality. Li^20^ proposed an occlusion-aware grasping method based on binocular stereo-vision. GCN is used to process occlusion relationships. The posture estimation module consists of PointNet + + point cloud feature extraction and GraspNet grasping posture prediction. However, the structural disorder, sparsity, and irregularity of point cloud data significantly increase the complexity of network architecture design and adversely affect the efficiency and stability of feature extraction.

Current research primarily emphasizes local feature descriptors for point clouds. Johnson and Hebert^21^ proposed the Spin Image (SI), one of the most widely cited 3D descriptors. However, SI has limited descriptive power and is sensitive to data resolution. Rusu^22^ introduced the Point Feature Histogram (PFH) descriptor, which is founded on the statistical deviation of angles between point normals. While PFH demonstrates significant discriminative power, it also entails considerable computational costs. To enhance efficiency, they subsequently proposed an enhanced version known as the Fast Point Feature Histogram (FPFH)^23^. FPFH primarily depend on the local geometric characteristics of the neighborhood, which makes it less robust in scenarios involving data occlusion or sparse point clouds. Dong^24^ introduced feature descriptors into the traditional TrICP algorithm for local feature extraction and used eigenvalues to represent local features. Li^25^ proposed a two-stage point cloud ground extraction framework UE-Extractor, aiming at improving the accuracy and efficiency of path planning for autonomous vehicles in complex terrains.

Although these descriptors have achieved substantial progress, most of them incur high computational complexity, thereby hindering the processing speed for large-scale point clouds. In addition, some feature extraction methods exhibit a marked decline in effectiveness under substantial noise interference, which impairs point cloud registration accuracy. As a result, few descriptors have achieved an optimal balance between robustness and efficiency. Due to their strong global search capabilities and low sensitivity to initial conditions, Swarm intelligence algorithms (SI) have been widely applied to complex optimization problems in recent years. Singh^26^ proposed an improved Follow The Leader (FTL) algorithm, which enhances optimization performance in truss structure design by simulating the behavior of sheep following a leader during foraging. Mehta^27^ proposed the MOBBO algorithm, which effectively solves multi-objective constrained optimization problems by simulating the behavior of brown bears. SI—such as Particle Swarm Optimization (PSO), Ant Colony Optimization (ACO), and Grey Wolf Optimizer (GWO)—offer global search capabilities in high-dimensional spaces, along with strong convergence and scalability. As a result, combining feature extraction techniques with conventional optimization methods has emerged as a promising strategy for enhancing the precision and efficiency of point cloud registration.

This study proposed a new local feature point extraction method and developed a point cloud registration algorithm based on the improved gray wolf optimizer. This feature extraction algorithm encloses the point cloud within a sphere centered at its centroid. The spherical surface is then partitioned into near-equal-area regions, and the points are projected onto it. Each projected point is assigned corresponding spherical coordinates and a surface-region label. Next, principal component analysis (PCA) is applied to the points within each region to compute their curvature variation. A nonlinear sampling weight is then assigned based on this variation, facilitating feature point extraction. Unlike methods that require computing curvature at every point, our approach uses region-based curvature variation as the feature descriptor, thereby reducing computational complexity. Two sets of experiments were conducted on three public datasets, focusing on feature point extraction and point cloud registration via the improved Grey Wolf Optimizer. The results show that our method extracts feature points effectively and achieves accurate registration. The main contributions of this paper are as follows:

1. A novel local feature point extraction method is presented. By introducing a region-based partitioning strategy to replace per-point operations on the entire point cloud, the proposed approach effectively reduces computational overhead and significantly shortens processing time, even for complex point cloud structures.
2. An improved Grey Wolf Optimizer-based registration algorithm is proposed. It achieves faster convergence while maintaining higher accuracy, effectively estimating the optimal rigid transformation parameters and significantly enhancing overall precision and applicability in complex scenarios.

## The proposed method of feature description and extraction

### Local area division of point clouds

In this study, we propose dividing the point cloud into distinct regions and analyzing curvature variation within each region to enable rapid and efficient extraction. The method presented herein involves partitioning the sphere into specific regions. Therefore, we use the centroid of the point cloud as the center of the sphere and determine the maximum distance from any point to this center as the radius. Any point on a sphere can be represented by two angles, *θ* and *ϕ*, where *θ* denotes the longitude with a range of $\left[ {0,2\pi } \right]$ and *ϕ* represents the latitude with a range of $\left[ {0,\pi } \right]$.

Divide the latitude *ϕ* into *n* segments as follows:

1 $${\phi _i}=\arccos \left( {1 - \frac{{2i}}{n}} \right),i=0,1, \ldots ,n.$$

The longitude *θ* is segmented, and given that it is uniformly distributed across the spherical surface, it can be effectively divided using an equally spaced method. The longitude interval $\left[ {0,2\pi } \right]$ is divided into *n* segments according to the following equation:

2 $${\theta _j}=\frac{{2\pi j}}{n},j=0,1, \ldots ,n.$$

According to the above partition, the sphere will be divided into ${n^2}$ grids. We project the points from the point cloud onto the spherical surface and determine the area corresponding to these projections based on the latitude and longitude coordinates of each projection point.

### Feature extraction

In point cloud processing, feature points are typically defined as locations where significant changes in the geometric properties of an object’s surface occur, such as edges, sharp corners, surface junctions, etc^28^. These locations generally exhibit high curvature values, whereas the curvature values of flat surfaces tend to be close to zero^29^. To further characterize the local geometric properties of a point, the eigenvalues of the covariance matrix and their corresponding eigenvectors are commonly used as descriptive indicators. This is because eigenvalues exhibit good rotational invariance under Euclidean transformations and effectively reflect the spatial distribution of points within their local neighborhood. PCA-based methods are computationally simple and can effectively extract representative geometric information from the neighborhood structure of both dense and sparse point clouds, making them widely used in point cloud processing tasks^30^. This paper proposes a method for extracting local features from point cloud data based on the rate of curvature change across different spherical regions. The core idea is to identify feature points with significant geometric variation by analyzing curvature changes across different local areas. The specific steps are as follows.

Principal Component Analysis (PCA) is used to calculate the rate of change in curvature for each patch, which is defined as the regions after the spherical region is divided. Instead of performing PCA on every individual point, which is computationally intensive and sensitive to noise, our method operates on grouped patches. By aggregating local geometric information within each patch, we can efficiently characterize structural variation in a more robust and stable manner. For patches containing more than 3 points, we extract the 3D coordinates of all points within the area and denote them as $P=\left\{ {{p_1},{p_2}, \ldots ,{p_N}} \right\}$. Subsequently, we calculate the covariance matrix Σ, as follows:

3 $$\sum =\frac{1}{{N - 1}}\sum\limits_{{i=1}}^{N} {\left( {{p_i} - \overline {p} } \right)} {\left( {{p_i} - \overline {p} } \right)^T}$$

4 $$\overline {p} =\frac{1}{N}\sum\limits_{{i=1}}^{N} {{p_i}} .$$

Here, $\overline {p}$ represents the mean vector of all points in the region, serving as an indicator of the center of gravity for that area; *N* denotes the number of points.

Perform eigenvalue decomposition on the covariance matrix to derive its three eigenvalues $\left\{ {{\lambda ^1}_{i},{\lambda _i}^{2},{\lambda _i}^{3}} \right\}$, which are arranged in ascending order. We define the curvature ${C_i}$ as the ratio of the smallest eigenvalue to the sum of all eigenvalues, as illustrated in the following formula:

5 $${C_i}=\frac{{\lambda _{i}^{1}}}{{\lambda _{i}^{1}+{\lambda _i}^{2}+\lambda _{i}^{3}}}.$$

This patch-based PCA strategy significantly reduces computational complexity while preserving essential geometric features, thereby overcoming the inefficiency typically associated with point-wise curvature estimation. Moreover, it enhances the stability of feature extraction by mitigating the influence of local outliers or irregular sampling.

To emphasize the characteristics of high-curvature regions, this paper employs a power transformation on the normalized curvature. The formula for this transformation is presented as follows:

6 $${W_i}={\left( {{C_i}^{\prime }+\varepsilon } \right)^\alpha },$$

where ${W_i}$ represents the transformed curvature weight, *ε* is a very small constant employed to prevent numerical instability that may arise from extremely small or zero curvature values, and *α* denotes the parameter that regulates the degree of curvature enhancement. When $\alpha >1$, regions exhibiting higher curvature values will be significantly amplified, thereby highlighting the geometric features of high-curvature areas. In this study, the parameter $\alpha >1$ is set to 2.5.

Based on the weight ${W_i}$ assigned to each region, the number of sample points allocated to that region can be determined. High-curvature regions are designated a greater number of sample points to preserve more detailed information. The total number of sample points is denoted as *P*, and the number of sample points ${N_{ij}}$ for each region $\left( {i,j} \right)$ can be calculated using the following formula:

7 $${N_i}={W_i} \cdot P.$$

The sampled points extracted from each region are used as the initial population for the optimization algorithm. This setup provides a reliable starting point for the optimization process, improving its convergence efficiency and stability to some extent, thereby laying a solid foundation for the accuracy of subsequent point cloud registration.

## Improved Grey Wolf Optimization algorithm (IGWO)

### Grey Wolf Optimization (GWO)

The essence of point cloud registration is to determine a rigid transformation matrix that aligns the source point cloud with the target point cloud through spatial rotation and translation. In three-dimensional space, such a transformation is defined by six parameters—three for rotation and three for translation. To achieve optimal alignment, these parameters must be accurately estimated. This process is essentially a complex global optimization problem. Choosing a suitable optimization algorithm is crucial to improving the accuracy and efficiency of registration. Common optimization methods currently include reinforcement learning (RL) and multi-criteria decision making (MCDM). RL^31^ excels in environments that require long-term strategy learning and interactive feedback. It optimizes transformation parameters through trial-and-error mechanisms, but its training process is typically complex and its generalization ability is limited when sample sizes are small. MCDM focuses on achieving an optimal trade-off among multiple objectives^32^. However, in high-dimensional continuous spaces, MCDM methods often lack global search capabilities and are prone to local optima. SI algorithms have been widely used in point cloud registration for their ability to perform efficient global searches by simulating collective behavior. They are particularly well-suited for high-dimensional, nonlinear, and gradient-free problems, and have become a key method for estimating rigid transformation parameters. Tejani^33^ compared the performance of GWO, SFS, and JADE in the context of steel frame design, and demonstrated that GWO exhibits superior global optimization ability for structural design. This provides theoretical support for applying GWO to point cloud registration and highlights the cross-domain applicability of such algorithms.

GWO is a swarm intelligence algorithm inspired by the social behaviors and predation strategies of grey wolves, as proposed by Mirjalili et al. in 2014^34^. The algorithm effectively simulates the hierarchical social structure and hunting techniques exhibited by grey wolves, including encircling, chasing, and attacking prey. In GWO, individuals within the population are categorized into four distinct levels: leaders (alpha), secondary leaders (beta), tertiary leaders (delta), and followers (omega). Throughout the optimization process, each wolf’s position represents a potential solution. Other individuals dynamically adjust their positions in response to the positions of the leaders, thereby simulating the process of encircling prey. This mechanism facilitates a gradual convergence towards the global optimum.

The pseudocode for the GWO algorithm is presented as follows:

**Algorithm 1:**
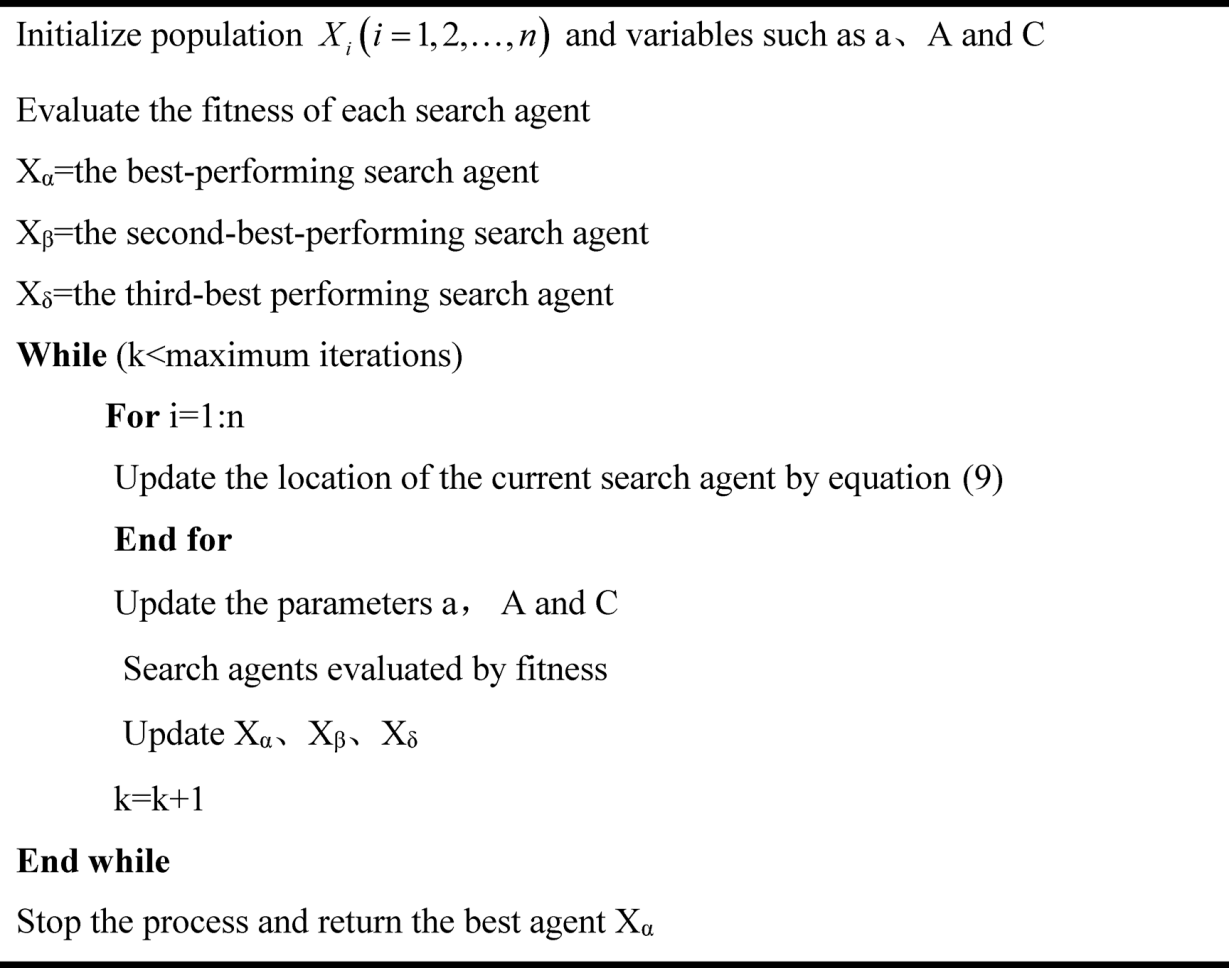
Grey Wolf Optimization algorithm.

In high-dimensional and complex optimization problems, the Grey Wolf Optimization (GWO) algorithm may experience premature convergence, resulting in the algorithm falling into local optima. This phenomenon adversely affects the overall performance of the optimization process. Furthermore, the Grey Wolf Optimizer (GWO) exhibits limitations in maintaining population diversity due to the absence of mechanisms such as crossover and mutation. This deficiency can lead to a decline in its global search capability. To address these challenges, this paper proposes a nonlinear adjustment of the convergence factor$\alpha$ and introduces a synchronous perturbation random approximation strategy. These enhancements aim to mitigate premature convergence and improve global search performance in the later stages of the optimization process.

### Nonlinear adjustment of the convergence factor *α*

In the GWO algorithm, the equation governing an individual grey wolf tracking the location of its prey is articulated as follows:

8 $$\left\{ \begin{gathered} {D_\alpha }=\left| {{C_1} \cdot {X_\alpha } - X} \right| \hfill \\ {D_\beta }=\left| {{C_2} \cdot {X_\beta } - X} \right| \hfill \\ {D_\delta }=\left| {{C_3} \cdot {X_\delta } - X} \right| \hfill \\ \end{gathered} \right.,$$

where ${D_\alpha },{D_\beta }$ and ${D_\delta }$ represent the distance between $\alpha ,\beta ,\delta$ and other individuals, respectively; ${X_\alpha },{X_\beta }$ and ${X_\delta }$ denote the current positions of $\alpha ,\beta$ and δ, respectively; ${C_1},{C_2}$ and ${C_3}$constitute a random vector. Additionally, *X* signifies the current position of the grey wolf.

9 $$X(t+1)=\frac{{{X_\alpha } - {A_1} \cdot {D_\alpha }+{X_\beta } - {A_2} \cdot {D_\beta }+{X_\delta } - {A_3} \cdot {D_\delta }}}{3}.$$

The final position of the *ω* individual is defined by Eq. (9). ${A_1},{A_2}$ and${A_3}$ are utilized to regulate the distance between the current position and the target prey position. The convergence factor *α* primarily influences the position update coefficient *A* for the wolf pack, as illustrated in Eq. (10). The convergence factor *α* decreases gradually with an increasing number of iterations *t*, and its expression is given as follows:

10 $$A=2a \cdot r - a$$

11 $$a=2 - \frac{{2t}}{{{T_{\hbox{max} }}}},$$

where *r* represents a random number within the range [0, 1], and ${T_{\hbox{max} }}$ denotes the maximum number of iterations.

In the initial phases of the algorithm, the convergence factor *α* is approximately 2, which permits the value of A to vary significantly within a large range of [− 2, 2]. This flexibility facilitates larger position updates for the wolf pack, thereby encouraging extensive exploration of the solution space and effectively avoiding premature convergence to local optima. As the algorithm advances, the convergence factor *α* gradually diminishes to 0, leading A to approach the interval of [− 1, 1]. At this juncture, the position updates of the wolf pack become increasingly smaller, concentrating more on fine local exploitation. This refinement facilitates the algorithm converge towards a more precise optimal solution.

However, the linearly decreasing convergence factor *α* is insufficient for effectively balance global search and local search capabilities^35^. To address this issue, this paper proposes a nonlinear improvement to the process of decreasing the convergence factor, with the new expression presented as follows:

12 $$a=\frac{2}{{1+{{\left( {\frac{t}{{{T_{\hbox{max} }} - t}}} \right)}^{2.5}}}}.$$

**Fig. 1:**
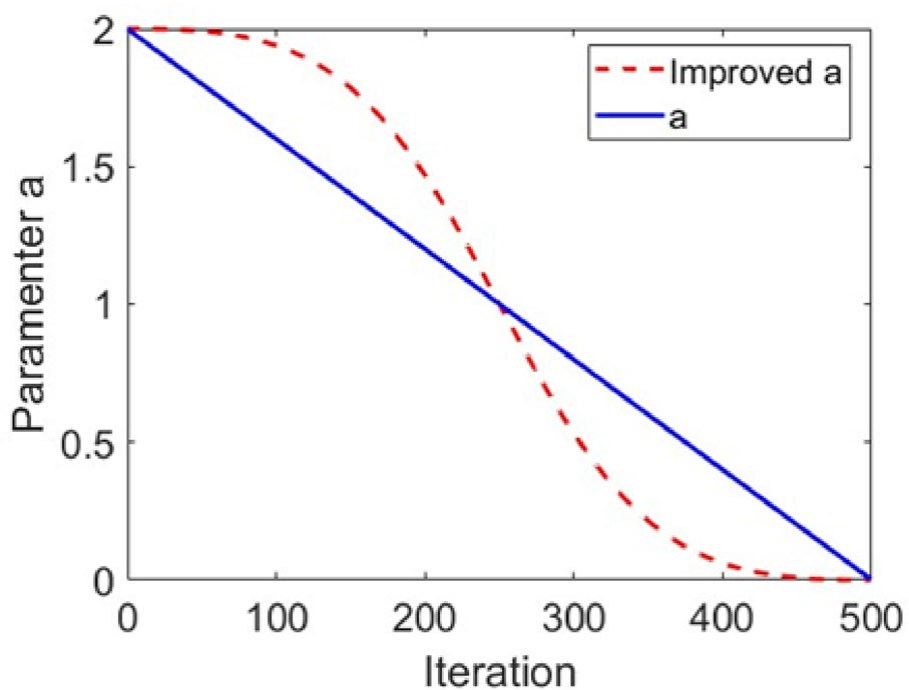
Change curves of the convergence factor *α*.

As illustrated in Fig. 1, the enhanced convergence factor adopts a nonlinear decay strategy that allows it to retain relatively larger values during the early iterations of the algorithm. This design promotes extensive exploration of the search space, thereby enhancing the algorithm’s global search capability and reducing the risk of premature convergence to local optima. As the iterations progress, the convergence factor gradually decreases to smaller values, which shifts the algorithm’s behavior from global exploration to local exploitation. This transition facilitates more precise fine-tuning of candidate solutions around the promising regions identified earlier. Compared to the original linear convergence factor used in standard GWO, the nonlinear variant enables a more adaptive balance between exploration and exploitation.

### Simultaneous perturbation stochastic approximation (SPSA)

The SPSA^36^ algorithm exhibits robust local search capabilities and demonstrates effective adaptability to multivariable optimization challenges. In this study, SPSA is incorporated into the position update process of the GWO algorithm, enabling the global search phase to fully capitalize on GWO’s exploration strengths while simultaneously combining the local search advantages of SPSA to attain higher-quality solutions. The primary concept is outlined as follows: the GWO algorithm is used for global optimization of the population, followed by the application of the SPSA algorithm to conduct a local search on the *α* wolf positions derived from the GWO algorithm. This two-step approach further refines the solution and enhances GWO’s capability to escape from local optima. The objective function is$f\left( {} \right)$, then in the SPSA algorithm, the gradient approximation formula during iterations is:

13 $${g_t}(\theta )=\frac{{f({X_i}(t)+{c_t} \cdot {\Delta _t}) - f({X_i}(t)+{c_t} \cdot {\Delta _t})}}{{2{c_t}}} \cdot {\Delta _t}^{{ - 1}}$$

14 $${c_t}=\frac{c}{{{{(t+1)}^\gamma }}},$$

Where *t* represents the current iteration number, while *c* and *r* are coefficients. Additionally, ${\Delta _t}$ denotes a d-dimensional random perturbation vector.

By integrating the gradient function, the updated position update mechanism for the grey wolf *α* is articulated as follows:

15 $${X_i}(t+1)={X_i}(t) - {a_t} \cdot {g_t}(\theta )$$

16 $${a_t}=\frac{a}{{{{(A+t+1)}^\alpha }}},$$

Where *α*, *A*, and *α* are coefficients. In this study, empirical values are used: $\alpha =0.602,\gamma =0.101,A=100,a=0.775,c=0.1$.

During each iteration of the update process, the value of the fitness function is computed. According to the greedy criterion, we recorded the better solution. The final position update formula for the grey wolf $\alpha$ is presented as follows:

17 $${X_i}(t+1)=\left\{ {\begin{array}{*{20}{c}} {{X_i}(t)}&{f({X_i}(t))<f({X_i}(t) - {a_i} \cdot {g_i}(\theta ))} \\ {{X_i}(t+1)}&{else} \end{array}} \right..$$

The improved GWO algorithm significantly enhances search efficiency and registration accuracy by introducing a nonlinear convergence factor strategy and the SPSA strategy. Specifically, the nonlinear convergence factor strengthens global exploration in the early stages, enabling more effective coverage of complex solution spaces, while the SPSA strategy improves local search precision in the later stages, facilitating accurate estimation of rigid transformation parameters. Through the synergy of these two strategies, the GWO algorithm demonstrates greater robustness and accuracy in point cloud registration tasks, particularly under challenging conditions such as low overlap or large pose deviations.

### Objective function and evaluation metrics

In the optimization process of GWO, the design of the objective function plays a critical role^37^. A well-defined objective function can effectively evaluate the alignment quality between point clouds and guide the search toward the global optimum. In this study, the six parameters to be optimized are defined as three rotation parameters $\alpha ,\beta ,\gamma$ and three translation parameters ${T_x},{T_y},{T_z}$. We define the original point cloud as $P=\left\{ {{p_1},{p_2},...{p_i}} \right\}$, while the point cloud to be registered is denoted as $Q=\left\{ {{q_1},{q_2},...{q_i}} \right\}$. The transformation matrix *T* between the two point clouds can be expressed as follows:

18 $$T={R_x} \cdot {R_y} \cdot {R_z} \cdot S$$

$${R_x}=\left( {\begin{array}{*{20}{c}} 1&0&0&0 \\ 0&{\cos \alpha }&{ - \sin \alpha }&0 \\ 0&{\sin \alpha }&{\cos \alpha }&0 \\ 0&0&0&1 \end{array}} \right){R_{y=}}\left( {\begin{array}{*{20}{c}} {\cos \beta }&0&{\sin \beta }&0 \\ 0&1&0&0 \\ { - \sin \beta }&0&{\cos \beta }&0 \\ 0&0&0&1 \end{array}} \right){R_z}=\left( {\begin{array}{*{20}{c}} {\cos \gamma }&{ - \sin \gamma }&0&0 \\ {\sin \gamma }&{\cos \gamma }&0&0 \\ 0&0&1&0 \\ 0&0&0&1 \end{array}} \right)S=\left( {\begin{array}{*{20}{c}} 1&0&0&{{T_x}} \\ 0&1&0&{{T_y}} \\ 0&0&1&{{T_z}} \\ 0&0&0&1 \end{array}} \right)$$

This paper uses the Root Mean Squared Error (RMSE) of point cloud registration as the objective function for Grey Wolf Optimization, as illustrated in Eq. (19). In each iteration of the update process, the fitness function value is computed, and the superior solution is recorded based on the greedy criterion.

19 $$f\left( {\alpha ,\beta ,\gamma ,{T_x},{T_y},{T_z}} \right)=\sqrt {\frac{1}{N}\sum\limits_{{i=1}}^{N} {{{\left\| {P \cdot T - Q} \right\|}^2}} }.$$

Denote the transformed point cloud be denoted as ${Q^\prime}$. To assess the accuracy of the point cloud registration, this paper calculates the Root Mean Squared Error (RMSE) distance between the original point cloud P and the transformed point cloud ${Q^\prime}$, as illustrated in the following formula:

20 $$RMSE=\sqrt {\frac{1}{N}{{\left\| {P - {Q^\prime}} \right\|}^2}},$$

Where *N* is the size of the source point cloud; Theoretically, a lower root-mean-square error (RMSE) indicates better point cloud alignment. In this study, the average RMSE and average registration time for all pairwise point clouds within each model are used to evaluate the performance of the proposed registration algorithm.

## Experimental results and analysis

### Test function experiments

**Fig. 2:**
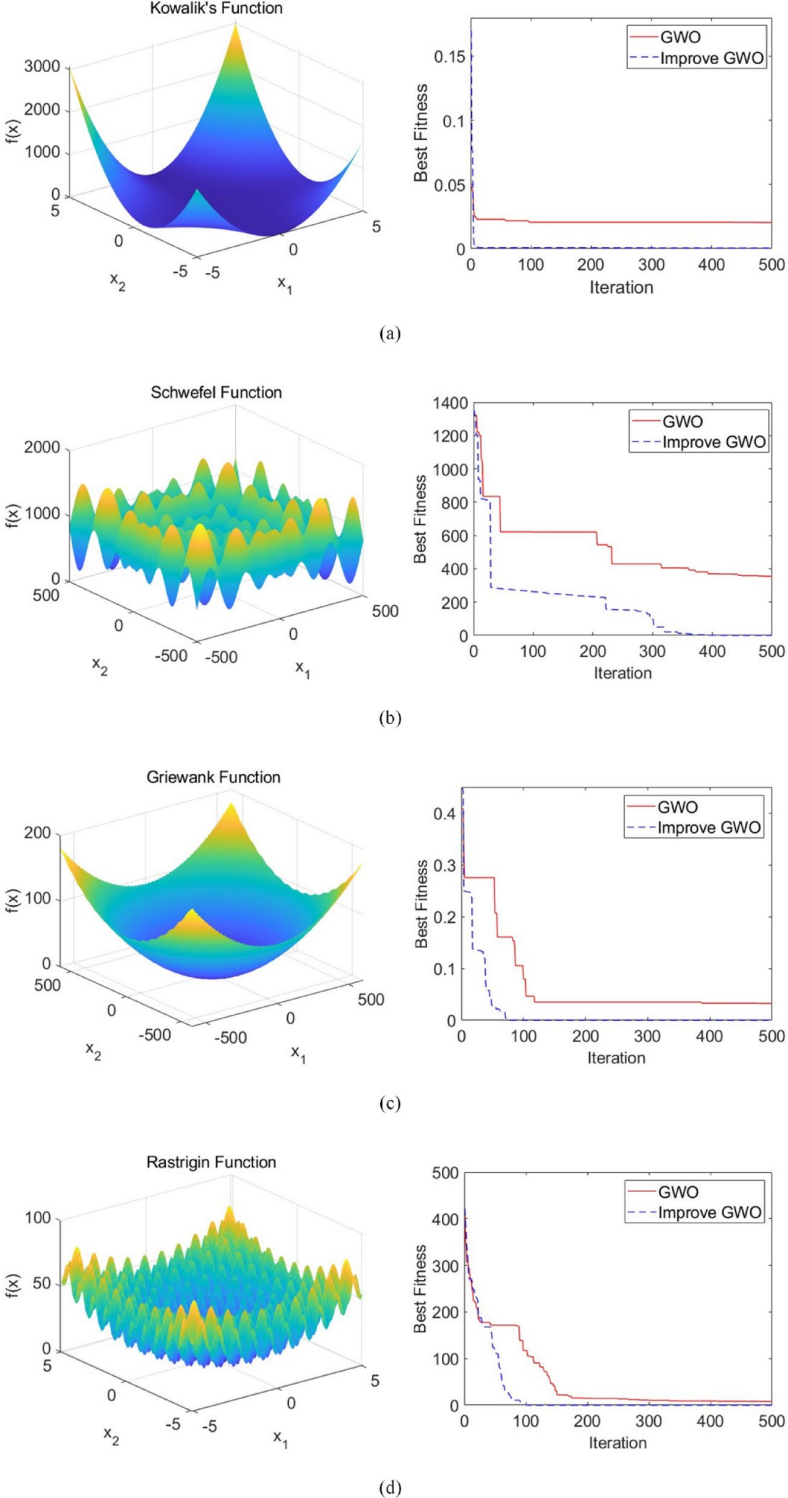
Function plots and convergence curves. **(a)** Kowalik’s function, **(b)** Schwefel function, **(c)** Griewank function, **(d)** Rastrigin function.

The optimization performance of the improved Grey Wolf Optimization (IGWO) algorithm was evaluated through a comparison analysis conducted between the original GWO algorithm and its improved version. Four test functions were selected for this study, as detailed in Table 1. The corresponding function plots and convergence curves are presented in Fig. 2. The convergence curves indicate that the improved GWO exhibits a significantly faster convergence trend across all four test functions. Compared to the original GWO, it achieves a rapid reduction in fitness value during the early iterations, demonstrating superior global search capability. Additionally, in the later stages of optimization, the improved GWO consistently attains a lower best fitness value, suggesting that the proposed modifications effectively enhance local search ability and improve solution accuracy.

**Table 1:** Test functions.

Function	Description	D	Range
Kowalik	$f\left( x \right)={\sum\limits_{{i=1}}^{{11}} {\left[ {{a_i} - \frac{{{x_1}\left( {b_{i}^{2}+{b_i}{x_2}} \right)}}{{b_{i}^{2}+{b_i}{x_3}+{x_4}}}} \right]} ^2}$	4	$\left[ { - 5,5} \right]$
Schwefel	$f\left( x \right)=418.9829D - \sum\limits_{{i=1}}^{D} {{x_i}} \sin \left( {\sqrt {\left| {{x_i}} \right|} } \right)$	6	$\left[ { - 500,500} \right]$
Griewank	$f(x)=1+\frac{1}{{400}}\sum\limits_{{i=1}}^{D} {x_{i}^{2}} - \prod\limits_{{i=1}}^{D} {\cos \left( {\frac{{{x_i}}}{{\sqrt i }}} \right)}$	6	$\left[ { - 600,600} \right]$
Rastrigin	$f(x)=\sum\limits_{{i=1}}^{D} {\left[ {x_{i}^{2} - 10\cos \left( {2\pi {x_i}} \right)+10} \right]}$	30	$\left[ { - 5.12,5.12} \right]$

### Feature extraction experiments

To validate the effectiveness of the proposed feature point extraction algorithm, we compared it with several mainstream methods, including FPFH, ISS, and Harris3D. Each method was integrated into a unified framework with the improved GWO registration algorithm to evaluate the impact of different keypoint strategies on point cloud registration performance. For consistency, the number of feature points was fixed at 2000 and the maximum number of iterations was set to 500.

**Fig. 3:**
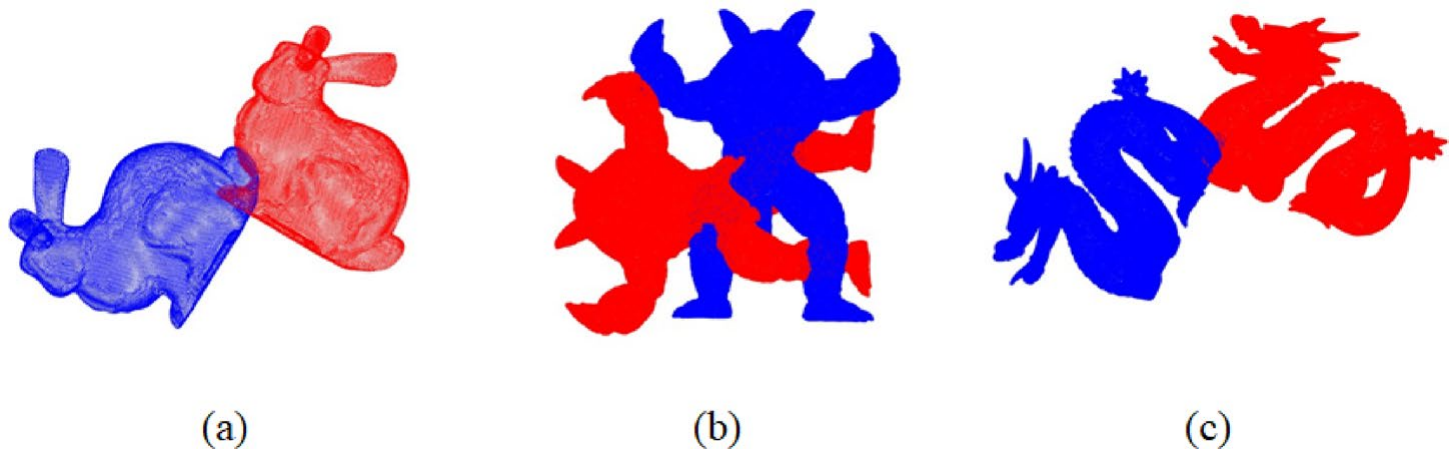
Initial state of the point clouds.

**Fig. 4:**
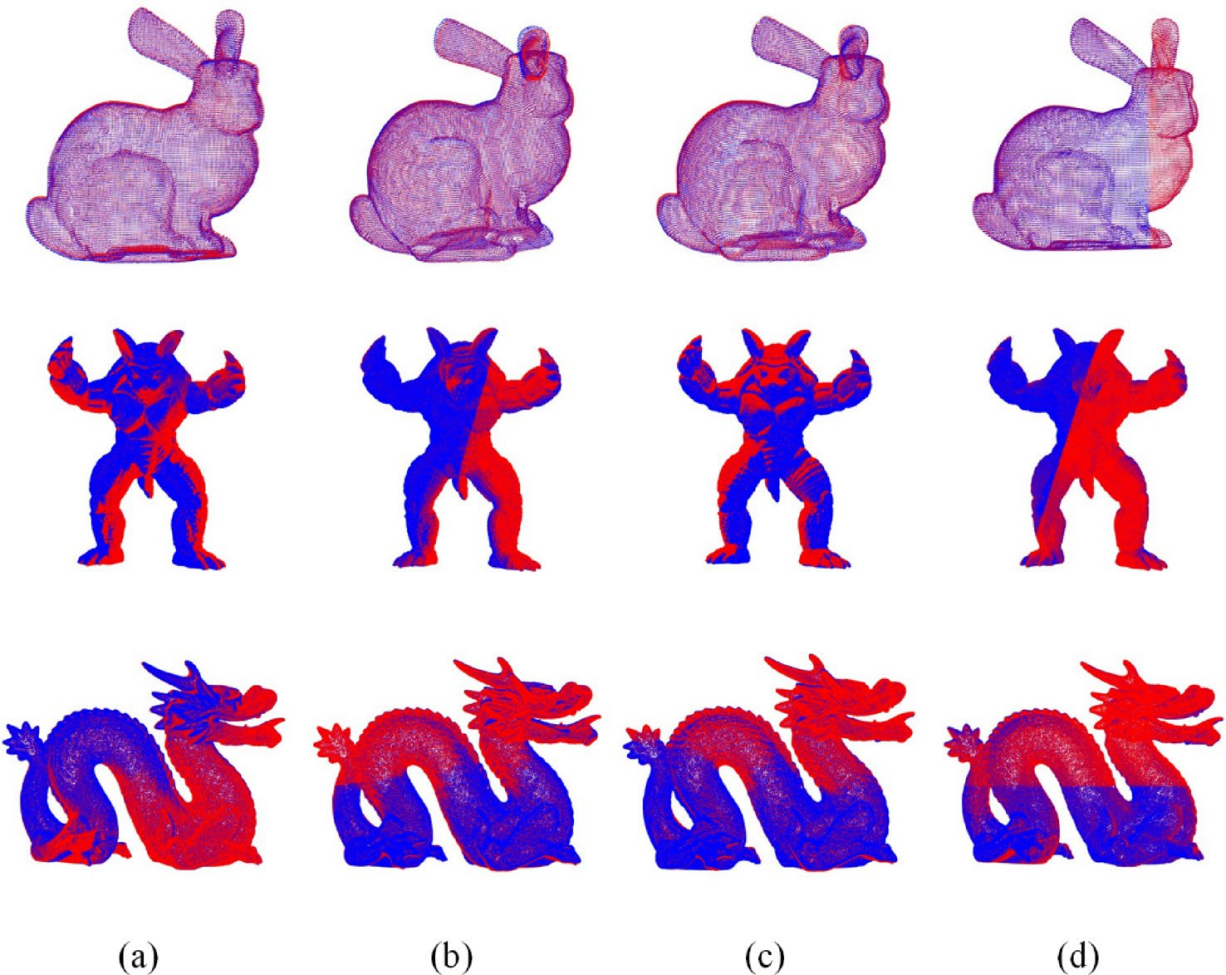
Registration results of different algorithms combined with the improved GWO. **(a)** Harris3D algorithm, **(b)** ISS algorithm, **(c)** FPFH algorithm, **(d)** Proposed algorithm. (© 2025 by Yichen Xie is licensed under CC BY 4.0. To view a copy of this license, visit https://creativecommons.org/licenses/by/4.0/)

The experiments were conducted on representative models from the Stanford 3D Scanning Repository, including Bunny, Armadillo, and Dragon. The Bunny, Armadillo, and Dragon point cloud datasets contain 35,947, 172,974, and 437,645 points respectively. The initial states of the models before registration are shown in the Fig. 3. Figure 4; Table 2 present the registration results using various feature extraction methods based on the improved GWO algorithm.

**Table 2:** Comparison of registration results of different feature extraction algorithms based on improved GWO.

Model	Harris3D		ISS		FPFH		Proposed	
	RMSE	Time (s)	RMSE	Time (s)	RMSE	Time (s)	RMSE	Time (s)
Bunny	0.00110	31.1	0.00096	17.7	0.00088	45.6	0.00028	19.8
Armadillo	0.64413	30.7	0.27552	19.3	0.49872	28.4	0.19391	20.3
Dragon	0.00045	44.6	0.00057	25.2	0.00106	65.2	0.00023	27.7

Traditional feature extraction algorithms often involve complex computations on all data points, resulting in long processing times and low efficiency when applied to large-scale point cloud datasets. The proposed method demonstrates superior temporal stability and computational efficiency. Although its processing time is slightly higher than that of ISS, it outperforms FPFH and Harris3D overall. It maintains fast response even on high-density point clouds, highlighting its scalability and practical value.

More importantly, the proposed method achieves a significant improvement in registration accuracy compared to traditional algorithms. Experimental results show that, relative to ISS, FPFH, and Harris3D, the registration accuracy improved by 64.6%, 53.4%, and 69.2%, respectively. This indicates stronger performance in feature descriptiveness and matching precision. While incurring a slight increase in processing time, the method yields substantial gains in accuracy. In summary, the proposed approach achieves an effective balance between efficiency and accuracy, offering a more robust and reliable feature extraction solution for point cloud registration tasks.

### Point cloud registration algorithm experiments

To evaluate the improvement in registration accuracy achieved by the proposed enhanced Grey Wolf Optimizer (GWO), two sets of comparative experiments were conducted. Both experiments were based on the previously introduced feature point extraction method, with 2000 keypoints uniformly extracted.

**Fig. 5:**
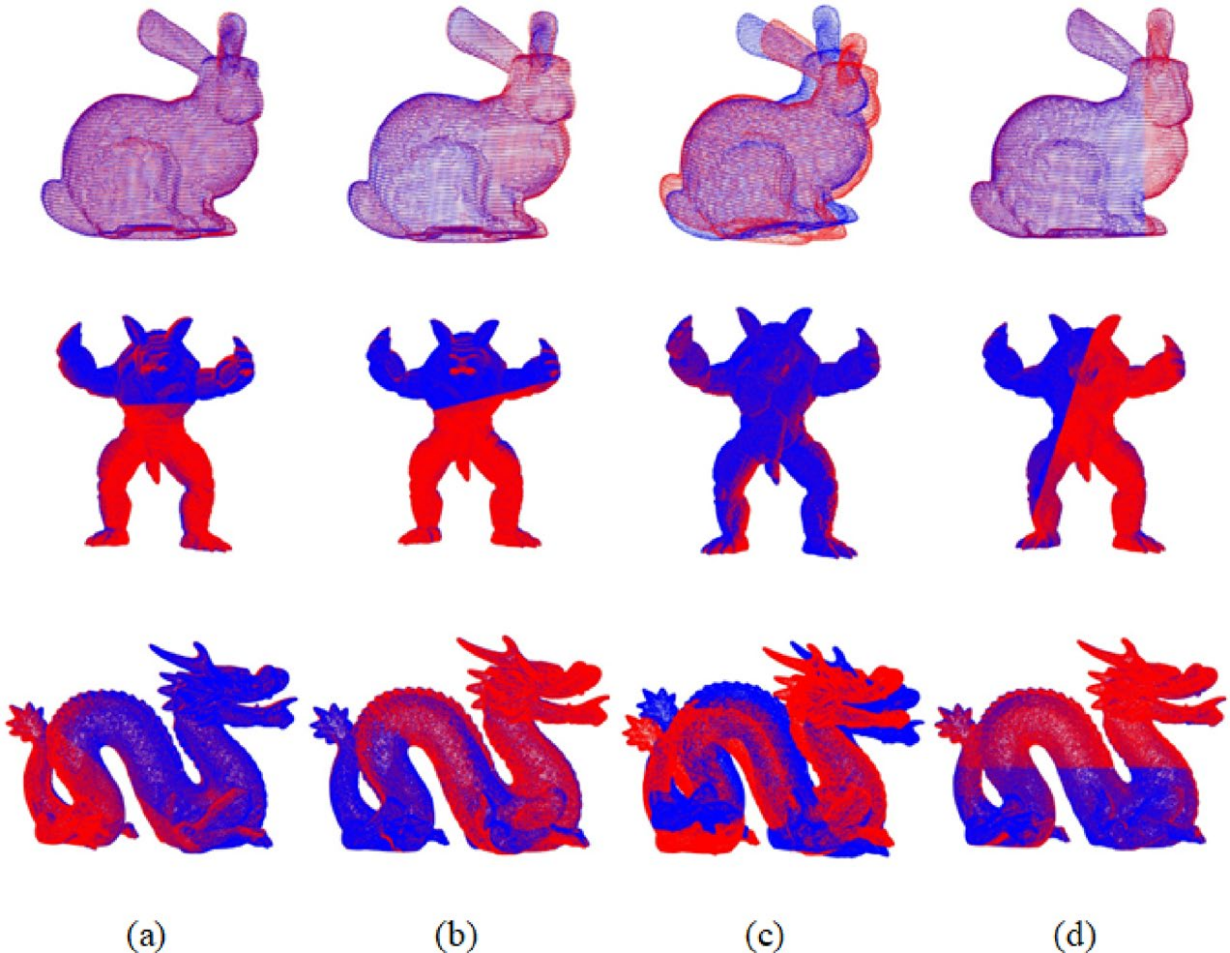
Registration results based on the proposed feature extraction method combined with different SI optimization algorithms. **(a)** PSO Algorithm, **(b)** GWO Algorithm, **(c)** HO Algorithm, **(d)** Improved GWO Algorithm.

In the first set of experiments, the improved GWO was compared with the traditional GWO, HO, and PSO. The results are presented in Fig. 5; Table 3.

**Table 3:** Comparison of registration results of different optimization algorithms based on the proposed feature extraction algorithms.

Model	PSO	GWO	HO	Improved GWO
	RMSE	RMSE	RMSE	RMSE
Bunny	0.00087	0.00069	0.00711	0.00028
Armadillo	0.24972	0.32787	0.23602	0.19391
Dragon	0.00031	0.00044	0.00559	0.00023

Experimental results show that the improved GWO algorithm outperforms the PSO, standard GWO, and HO algorithms in terms of registration accuracy, with respective improvements of 39.1%, 49.3%, and 68.6%. Consequently, the proposed feature extraction method, in conjunction with the improved GWO algorithm, exhibits superior registration accuracy relative to traditional optimization algorithms.

**Fig. 6:**
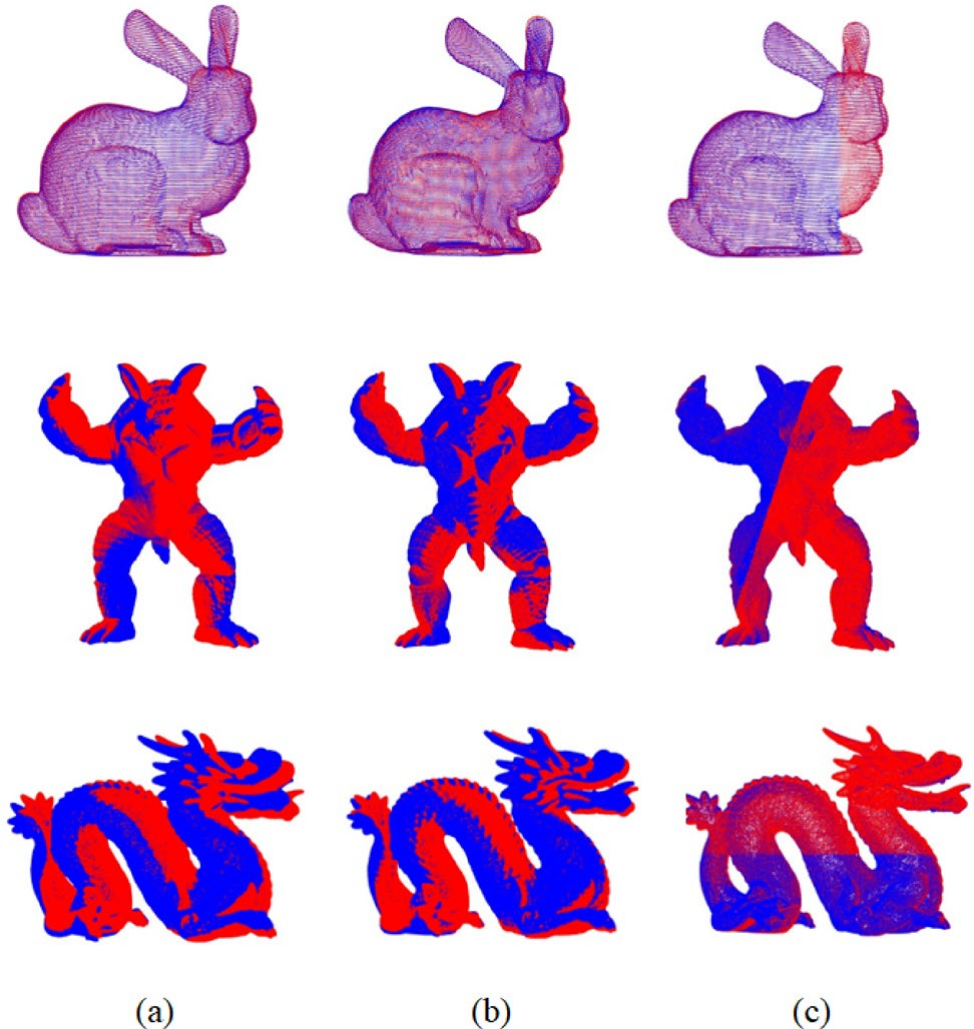
Registration results based on the proposed feature extraction method combined with different registration algorithms. **(a)** ICP Algorithm, **(b)** TrICP Algorithm, **(C)** Improved GWO Algorithm.

The second set of experiments further compared the improved GWO with the classical Iterative Closest Point (ICP) algorithm and TrICP. This set also employed the same keypoint extraction strategy with 2000 features. The corresponding results are shown in Fig. 6; Table 4.

**Table 4:** Comparison of registration results of different registration algorithms based on the proposed feature extraction algorithms.

Model	ICP	TrICP	Improved GWO
	RMSE	RMSE	RMSE
Bunny	0.00062	0.00069	0.00028
Armadillo	0.67203	0.74103	0.19391
Dragon	0.00372	0.00171	0.00023

The ICP algorithm estimates the rigid transformation by iteratively minimizing the distance between corresponding points in the source and target point clouds. Building upon this, the TrICP algorithm incorporates overlap estimation by rejecting inconsistent point pairs. As a result, traditional ICP is highly sensitive to the initial pose overlap and typically achieves satisfactory registration only under high-overlap conditions, whereas TrICP offers better adaptability in low-overlap scenarios (5). Experimental results show that the registration accuracy of ICP drops significantly when the initial overlap is low. In contrast, the proposed feature extraction method, combined with the improved GWO algorithm, demonstrates superior registration performance, with significantly higher accuracy compared to both ICP and TrICP. Although the proposed feature extraction method combined with the improved GWO algorithm demonstrates superior registration accuracy, its performance may still be affected by factors such as the precision of keypoint extraction, sensitivity to parameter settings, and susceptibility to local optima during the optimization process. These limitations are particularly evident when dealing with point clouds containing severe noise or ambiguous geometric structures, where instability may occur.

## Conclusion

This paper develops an improved feature point extraction approach that significantly improves registration accuracy and computational efficiency in complex point cloud processing. This approach incorporates regional curvature features to develop a surface feature descriptor of the point cloud, while employing the curvature variation rate to nonlinearly assign sampling weights. This enables rapid and efficient extraction of feature points across various point cloud models. Furthermore, this study integrates the point cloud registration process with intelligent optimization algorithms by employing the improved GWO algorithm to identify the optimal parameters for point cloud registration. The GWO algorithm enhances global search ability and local optimization accuracy through nonlinear convergence factor adjustment and integration of a synchronous perturbation stochastic approximation strategy. This paper presents experimental analysis utilizing three classic point cloud models from the Stanford dataset as test subjects. The results of the experiments indicate that the proposed feature extraction algorithm, in conjunction with the improved GWO algorithm, achieves greater accuracy and efficiency in registration.

## Data Availability

The data presented in this study are available on request from the corresponding author.
